# The Role of MicroRNA, Long Non-Coding RNA and Circular RNA in the Pathogenesis of Polycystic Ovary Syndrome: A Literature Review

**DOI:** 10.3390/ijms25020903

**Published:** 2024-01-11

**Authors:** Jenan Sh. Nasser, Noor Altahoo, Sayed Almosawi, Abrar Alhermi, Alexandra E. Butler

**Affiliations:** 1 School of Medicine, Royal College of Surgeons of Ireland, Busaiteen, Adliya 15503, Bahrain; 20202482@rcsi-mub.com (J.S.N.); 20204236@rcsi-mub.com (N.A.); 20204234@rcsi-mub.com (S.A.); 20205606@rcsi-mub.com (A.A.); 2 Research Department, Royal College of Surgeons of Ireland, Busaiteen, Adliya 15503, Bahrain

**Keywords:** polycystic ovary syndrome (PCOS), microRNA (miRNAs), infertility, cardiovascular disease, insulin resistance, circadian rhythm, biomarkers, therapeutic targets, long coding RNA (lncRNA), circular RNA (circRNA)

## Abstract

Polycystic ovary syndrome (PCOS) is the most common endocrine-metabolic disease in females of reproductive age, affecting 4–20% of pre-menopausal women worldwide. MicroRNAs (miRNAs) are endogenous, single-stranded, non-coding, regulatory ribonucleic acid molecules found in eukaryotic cells. Abnormal miRNA expression has been associated with several diseases and could possibly explain their underlying pathophysiology. MiRNAs have been extensively studied for their potential diagnostic, prognostic, and therapeutic uses in many diseases, such as type 2 diabetes, obesity, cardiovascular disease, PCOS, and endometriosis. In women with PCOS, miRNAs were found to be abnormally expressed in theca cells, follicular fluid, granulosa cells, peripheral blood leukocytes, serum, and adipose tissue when compared to those without PCOS, making miRNAs a useful potential biomarker for the disease. Key pathways involved in PCOS, such as folliculogenesis, steroidogenesis, and cellular adhesion, are regulated by miRNA. This also highlights their importance as potential prognostic markers. In addition, recent evidence suggests a role for miRNAs in regulating the circadian rhythm (CR). CR is crucial for regulating reproduction through the various functions of the hypothalamic-pituitary-gonadal (HPG) axis and the ovaries. A disordered CR affects reproductive outcomes by inducing insulin resistance, oxidative stress, and systemic inflammation. Moreover, miRNAs were demonstrated to interact with lncRNA and circRNAs, which are thought to play a role in the pathogenesis of PCOS. This review discusses what is currently understood about miRNAs in PCOS, the cellular pathways involved, and their potential role as biomarkers and therapeutic targets.

## 1. Introduction to Polycystic Ovary Syndrome

Polycystic ovary syndrome (PCOS) is the most common endocrine-metabolic disease in females of reproductive age [1]. It affects 4–20% of pre-menopausal women worldwide [2]. PCOS is a heterogeneous disorder, and the diagnosis is one of exclusion [3]. It is defined by a specific group of signs and symptoms that include ovarian dysfunction, polycystic ovaries, and hyperandrogenism [3]. Typical manifestations of hyperandrogenism in women include acne, male pattern hair loss, and hirsutism, with increased serum androgen concentration on laboratory testing [4]. Other features of the disease include irregular menstrual periods, infertility, and the typical polycystic appearance of the ovaries on transvaginal ultrasound (TVUS). PCOS patients can also experience mood disorders, including anxiety and depression, eating disorders, and impaired quality of life [4]. About 40–85% of women with PCOS are overweight or obese, with insulin resistance present in ~30% of lean women and 70% of obese women with PCOS [5]. Thus, women suffering from PCOS have an increased risk of developing health-related problems such as diabetes and cardiovascular disease [5].

PCOS has an ill-defined etiology. However, its pathogenesis can be envisioned as a “two-hit” phenomenon, with the first hit being genetic predisposition [6] and the second being factors such as insulin resistance, hyperinsulinism, and obesity [7]. The clustering of cases within families indicates that PCOS does have a genetic component; however, the exact mode of inheritance is elusive and most likely to be polygenic and multifactorial [8]. As PCOS women have reduced fertility, this syndrome presents a puzzling genetic paradox with regard to natural selection, as genes that are passed down are either positive or neutral genes. One theory suggests that PCOS could have developed as a series of independent genetic mutations aiming to preserve anabolism during times of nutritional deprivation via increased androgens and insulin production [7,9]. As such, PCOS could be considered as a mismatch disorder characterized by a sedentary lifestyle, where drastic changes in physical activity, changes in diet, elevated exposure to hazardous pollutants, decreased pregnancies, as well as rapid urbanization could all push toward manifestation of this syndrome [9].

One of the difficulties in delineating the etiology of PCOS is that the criteria for diagnosis have varied. Currently, the most used diagnostic criteria were accepted by the European Society of Human Reproduction and Embryology/American Society for Reproductive Medicine (ESHRE/ASRM) in Rotterdam in 2003 [10]. According to this consensus, “PCOS is defined by the presence of two of three of the following criteria: oligo-anovulation, hyperandrogenism, and polycystic ovaries (≥12 follicles measuring 2–9 mm in diameter and/or an ovarian volume > 10 mL in at least one ovary)” [11]. Moreover, four main phenotypes of PCOS were identified based on the presence of androgen excess, ovulatory dysfunction, and polycystic ovaries on TVUS following the Rotterdam Criteria (Table 1). Notably, insulin resistance is the most common classical phenotype (found in types A and B) (80%), followed by ovulatory PCOS (type C) (65%) and non-hyperandrogenic PCOS (type D) [12].

In PCOS, the mechanisms underlying the common systemic manifestations of hyperandrogenism, insulin resistance, and obesity are poorly understood [7]. Hirsutism, the most common presentation of women with PCOS, is indicative of hyperandrogenism [13]. Other presenting symptoms include irregular or absent menses and infertility [14]. There is much still to be understood about the etiology of PCOS and to delineate why these presentations commonly lead to the same outcome. One area of active research is the role of microRNAs (miRNAs) in the pathogenesis of PCOS and their utility as potential biomarkers and therapeutic targets.

## 2. Methodology

Several databases were used to ensure that a high-quality literature search was conducted. Relevant articles were identified from inception until July 2023 in PubMed, Medline, Science direct, Embase, and Google Scholar using the following keywords: polycystic ovary syndrome (PCOS), microRNA (miRNAs), infertility, cardiovascular disease (CVD), insulin resistance, circadian rhythm, prognostic biomarkers, diagnostic biomarkers, therapeutic targets, long non-coding (lncRNA), circular RNA (circRNA). Only articles in English were included in the discussion. References cited in the identified articles were also examined on a case-by-case basis. Articles were excluded if they were published in a language other than English or did not present original findings, e.g., letters to the editor and opinion pieces.

## 3. Introduction to microRNA

MiRNAs are a class of endogenous, single-stranded, noncoding ribonucleic acid molecules found in eukaryotic cells [15]. They are about 19 to 25 nucleotides in length and function to regulate post-transcriptional gene expression by binding to the 3′UTR of target mRNA to suppress or enhance its translation [15]. Binding to 5′UTR, gene promoters and coding sequences have also been described, suggesting an effect on both translation and transcription [16,17]. MiRNAs are generally divided into two classes according to their origin in the genome: those encoded in exons and those from overlapping introns, with around 50% being expressed from non-protein coding regions [18]. The expression of miRNA is affected by the same regulatory mechanisms of protein-coding genes, such as epigenetic regulation and a regulatory feedback loop [19,20]. Growing evidence suggests that these small molecules play a pivotal role in cell differentiation and proliferation, metabolism, apoptosis, stress responses, and cell-cycle regulation [21,22].

MiRNAs are processed via canonical or non-canonical pathways, the canonical pathway being predominant [17,18]. Key proteins involved in miRNA biogenesis in both pathways include the Drosha, Dicer, exportin-5, and Argonaute family proteins [17]. In the canonical pathway, the synthesis of miRNA is initiated in the nucleus by the binding of RNA polymerase II/III to DNA genes encoding for primary miRNA (pri-miRNA), either post- or co-transcriptionally [17]. A stem-loop structure of pri-miRNA is transcribed, which is then cleaved into a hairpin-structure precursor miRNA (pre-miRNA) by the enzyme Drosha. Pre-miRNA is transported to the cytoplasm through the exportin-5 complex to be processed by the enzyme Dicer-transactivation response RNA binding protein (TRBP) into a miRNA duplex [23]. Further structural processing of the miRNA duplex occurs to produce a mature miRNA [17,18]. Understanding these processes is crucial to developing therapeutic strategies targeting miRNAs [24,25].

Moreover, the study of miRNAs has provided a new perspective on the regulation of gene expression post-transcriptionally [26]. Abnormal miRNA expression has been associated with several human diseases, such as type 2 diabetes, obesity, cardiovascular disease, PCOS, endometriosis, and cancer [20,27,28,29,30]. In addition to cellular expression, miRNAs can also be found circulating in the plasma and serum [31]. They have also been detected in urine, semen, saliva, and microvesicles, making them useful potential diagnostic and prognostic markers of disease [19]. MiRNAs can be secreted outside cells, loaded into high-density lipoproteins, attached to Argonaute RISC catalytic component 2 (Ago2), or packaged in exosomes [26]. The release of exosomal miRNA in extracellular fluid is mediated by a ceramide-dependent pathway and is thought to have a growth regulatory effect on target cells [32].

Furthermore, miRNAs have been suggested to play a role in the onset and development of PCOS [33,34]. Under normal conditions, they are expressed in theca cells, follicular fluid, and granulosa cells and are considered important regulators of metabolic processes [33]. Key pathways regulated by miRNA include folliculogenesis, steroidogenesis, and cellular adhesion, each of which is hypothesized to be involved in the pathogenesis of PCOS [33]. In PCOS women, miRNAs were found to be abnormally expressed in theca cells, follicular fluid, granulosa cells, peripheral blood leukocytes, serum, and adipose tissue when compared to women without PCOS [33]. As such, miRNAs have been widely studied with the aim of understanding the pathogenesis of PCOS and identifying potential biomarkers for the disease [34]. Therefore, in this review, we summarize current evidence about the role of miRNAs in the pathogenesis of PCOS and its complications (infertility, insulin resistance, and cardiovascular disease), as well as their use as potential diagnostic and prognostic biomarkers. Furthermore, we discuss the role of the circadian rhythm in reproduction and its dysfunction in PCOS, together with the interactions between miRNAs and other RNAs in PCOS.

## 4. The Role of miRNA in the Pathogenesis of Common PCOS Complications

### 4.1. Infertility

**Figure 1:**
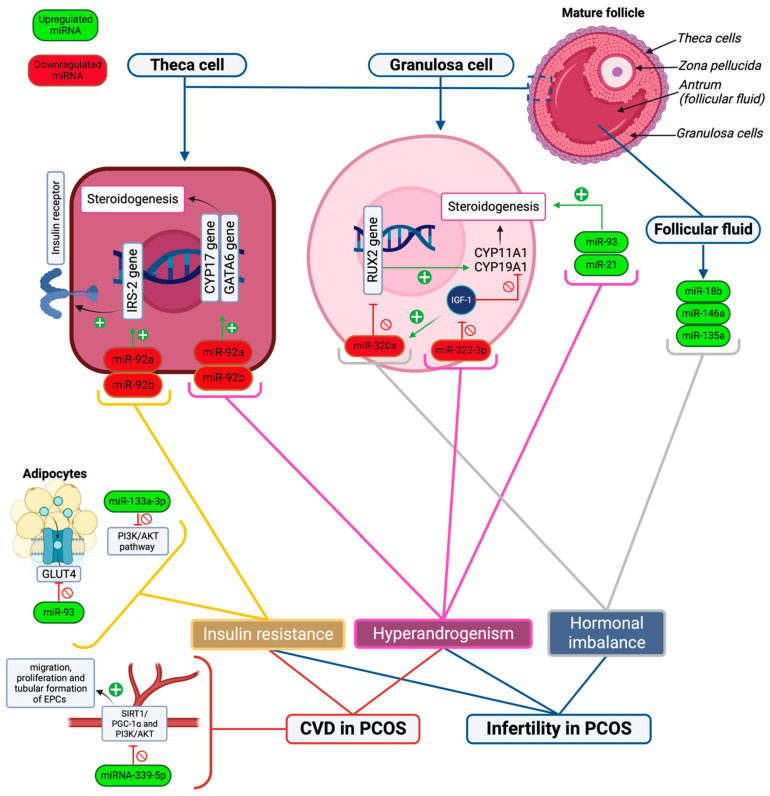
A simplified model showcasing the role of important miRNAs in the pathogenesis of PCOS complications. Upregulated miRNAs are shown in green font, and downregulated miRNAs are shown in red font. The main drivers of PCOS complications are insulin resistance, hyperandrogenism, and other hormonal imbalances. Infertility in PCOS is mediated by insulin resistance, hyperandrogenism, and hormonal imbalance, while CVD risk is affected by hyperandrogenism, insulin resistance, and reduced vascular endothelial health. The figure shows that the inhibition of the PI3K/AKT pathway in cells (due to the up regulation of miR-133a-3p), GLUT4 receptors (due to upregulation of miR-93), and increased expression of IRS-2 proteins (due to downregulation of miR-92a and -92b) play a role in insulin resistance in PCOS. Meanwhile, the downregulation of miR-323-3p, which reduces the activity of IGF-1, and upregulation of miR-93 and -21 in granulosa cells results in hyperandrogenism. Likewise, the downregulation of miR-92a and -92b in theca cells leads to hyperandrogenism by increasing the expression of *CYP17* and *GATA6* genes which stimulate androgen synthesis. Moreover, dysregulated levels of progesterone, estradiol, and testosterone are affected by the upregulation of miR-18b, -146a, and -135a in follicular fluid, resulting in hormonal imbalance. The downregulation of miR-320a also leads to hormonal variations by reducing the expression of the *RUX2* gene, which modulates steroidogenesis in granulosa cells. Additionally, miRNA-339-5p leads to vascular endothelial cell damage by inhibiting SIRT1/PGC-1α and PI3K/AKT signaling pathways, which inhibits EPC migration, tubular formation, and proliferation and contributes to the risk of CVD in PCOS. Created with BioRender.com.

PCOS is characterized by ovulatory dysfunction, making it more likely for patients to experience poor pregnancy outcomes. Women suffering from PCOS experience an alarming rate of infertility and are at risk of fetal, neonatal, and maternal complications [35,36]. The incidence of PCOS in women with anovulatory infertility is 70–80% [37]. Unsurprisingly, a significant number of PCOS women will undergo infertility treatment in order to conceive, with many commonly experiencing pregnancy complications such as pre-eclampsia, premature birth, and gestational diabetes [34,38]. Dysregulated miRNAs are thought to be important in the ovulatory dysfunction of patients suffering from PCOS and contribute to the high incidence of infertility among this patient group [39] (Table 2). Moreover, it is believed that there is an association between insulin resistance (IR) and hyperandrogenism that is directly linked to infertility problems in PCOS [40] (Figure 1).

Various microRNAs are significantly increased in PCOS women, causing hormonal imbalances that disrupt the menstrual cycle. Three of these microRNAs were identified to be overexpressed in the follicular fluid of PCOS patients compared to healthy controls. This includes miR-18b, miR-146a, and miR-135a [41]. MiR-18b promotes the release of progesterone while inhibiting the release of estradiol and testosterone, thus negatively impacting fertility. MiR-146a reduces progesterone, estradiol, and testosterone release, causing irregularities in the menstrual cycle. MiR-135a reduces progesterone and testosterone release [41].

It has also been demonstrated that hyperandrogenic PCOS patients have increased expression of miR-93 and miR-21 in comparison to normal androgenic PCOS patients. MiR-93 and miR-21 have been highlighted as androgen-dependent factors, as free androgen and free testosterone index positively correlated with them in granulosa cells of women with PCOS. This indicates that, under hyperandrogenic conditions, they may have a role in follicular dysfunction [42]. In addition, miRNAs have been identified as having an impact on relative estrogen deficiency. A significant imbalance in estradiol and androgen in granulosa cells has been related to miR-27a-3p [43]. Additionally, the downregulation of miR-320a in granulosa cells is thought to promote estrogen deficiency and impact insulin-like growth factor 1 (IGF-1) regulatory mechanisms [44]. This is mediated through the expression of CYP11A1 and CYP19A1 by direct targeting of the *RUNX2* gene [44].

MiRNAs also play an important role in ovarian theca cell function. It has been suggested that PCOS patients have downregulated expression of miR-92a and miR-92b in theca cells, which regulate 17-hydroxylase/C17–20 lyase cytochrome P50 (*CYP17*), GATA-binding factor 6 (*GATA6*), and insulin receptor substrate proteins 2 (IRS-2) expression [45]. This could play a role in androgen biosynthesis dysregulation in theca cells, as theca cells and granulosa cells usually produce non-steroidal factors that affect mutual differentiation and proliferation throughout folliculogenesis [23,45]. Moreover, miR-323-3p found in cumulus cells has also been proven to be dysregulated in PCOS patients compared to healthy controls [43]. It is thought that miR-323-3p directly binds to IGF-1 mRNA and inhibits steroidogenesis and cumulus cell apoptosis in women who do not suffer from PCOS. However, in PCOS patients, miR-323-3p is downregulated, leading to the upregulation of steroidogenesis and promotion of apoptosis, which indicates that it could have a significant role in the development of PCOS and infertility [43].

Overall, existing evidence supports an association between PCOS and miRNA dysregulation in granulosa cells and theca cells, which may contribute to PCOS evolution, hyperandrogenemia, and complications with regard to fertility. This implies that manipulation of miRNAs could have a role in improving follicular status and health in women with PCOS.

### 4.2. Insulin Resistance

Insulin resistance (IR) in PCOS is caused by impaired insulin action characterized by compensatory hyperinsulinemia (HI) and reduced insulin response to glucose overload. As such, IR in women alters several metabolic pathways in target organs, including, but not limited to, adipose tissue, skeletal muscle, liver, and brain. Androgen excess promotes a vicious cycle with IR and HI [46]. IR and compensatory HI are present in 65–95% of women diagnosed with PCOS and are exacerbated by obesity [46].

In women with PCOS, some miRNAs are increased in expression and associated with insulin resistance (Table 3). One such miRNA is miRNA-93, which was shown to be overexpressed and to correlate strongly with the downregulation of GLUT4 receptors in adipose tissue in women with PCOS [47]. However, it was also observed to be elevated in other IR-associated conditions such as Type 2 diabetes mellitus (T2DM) and obesity [47]. Another key miRNA is miRNA-146, which is closely associated with insulin resistance and is believed to play a key role in IR pathogenesis. Expression of this miRNA leads to a cascade of proinflammatory signals in which nuclear factor kappa B (NFkB) becomes activated, which increases miR-146a levels in order to institute negative feedback regulation and control of immune responses. During hyperglycemia, miR-146 levels may be decreased despite NFkB activation and proinflammatory responses, which could originate from pre-miR-146 polymorphisms [48]. Moreover, this miRNA could be useful as a diagnostic or therapeutic target, but this requires further investigation [48]. MiRNA-133a-3p was also more highly expressed in PCOS women compared to controls, with a further study demonstrating that, amongst PCOS subjects, obese PCOS women had the highest expression levels [48]. This miRNA inhibits the phosphoinositide-3-kinase/protein kinase B (PI3K/AKT(PKB)) signaling pathway, which is well-established for its importance in insulin action. Insulin activates PI3K through the insulin receptor substrate-1 to promote its main downstream molecular protein, AKT, which is activated by PI3K phosphorylation on ser473 and thr308. IR in PCOS leads to PI3K/AKT signaling inhibition. However, further studies are required to fully elucidate the mechanisms involved [48].

Furthermore, the administration of metformin has been linked with the altered expression of miR-222 and miR-221 in patients with T2DM [49]. Studies on dipeptidyl peptidase-4 (DPP-4) inhibitors and glucagon-like peptide 1 agonist receptor agonist (GLP-1 RA) noted a similar relationship, including the altered expression of miR-6763, miR-33, miR-155-5p, miR-6356, miR-197, miR-875-5P, and miR-1197-3p [50,51]. These possible effects of miRNA on insulin sensitivity may have a significant role in enhancing symptoms related to PCOS by increasing glucose metabolism and transport [52]. Furthermore, miR-143-3p and miR-155-5p have been shown to antagonize glycolysis in patients with PCOS-related follicular dysplasia. A study by Cao et al. demonstrated that follicular granulosa cells in patients with PCOS had high testosterone as well as a glucose-enriched environment. MiR-143-3p has been demonstrated to negatively regulate glycolysis, while miR-155-5p positively regulates glycolysis [53]. This provides two potential options that require further study as a treatment target in PCOS patients.

**Table 3:** MiRNAs involved in the pathogenesis of insulin resistance in women with PCOS.

miRNA(s)	Study Finding	References
miRNA-93	Upregulated in PCOS, resulting in the downregulation of GLUT4 receptors in adipose tissue.	[47]
miRNA-146	Role in insulin resistance and inflammatory modulation.	[48]
miRNA-133a-3p	Upregulation resulting in the inhibition of the PI3K/AKT signaling pathway that is important in insulin activity.	[48]
miR-222 miR-221	Altered expression with the administration of metformin in T2DM patients, leading to increased glucose metabolism and transport.	[49]
miR-26a	Altered expression with the administration of metformin for pancreatic stem cell markers.	[54]
miR-6763 miR-33 miR-155-5p miR-6356 miR-197 miR-875-5P miR-1197-3p	Altered expression with dipeptidyl peptidase-4 (DPP-4) inhibitors and glucagon-like peptide 1 agonist receptor agonist (GLP-1 RA), leading to increased glucose metabolism and transport.	[50]
miR-143-3p	Negatively regulates glycolysis.	[53]
miR-155-5p	Positively regulates glycolysis.	[53]

### 4.3. Cardiovascular Complications

There is conflicting evidence on the relationship between PCOS and cardiovascular disease (CVD) [55,56]. However, several studies have suggested that women with PCOS are at a higher risk of developing CVD due to the increased prevalence of obesity and insulin resistance in this population, leading to cardiometabolic dysfunction and an unfavorable metabolic profile [38,57]. This risk appears to be greater in women who present with hyperandrogenism [38,55]. In addition, subclinical CVD markers, such as endothelial dysfunction, coronary artery calcium scores, carotid intima-media thickness, and other markers, were more likely to be elevated in women with PCOS, indicating a possible risk for future cardiovascular events [57,58]. The increased oxidative stress associated with PCOS further increases this risk [59]. Nonetheless, the underlying pathogenesis of CVD in PCOS is still unclear, and several hypotheses have been proposed to explain it.

The role of miRNAs in the pathogenesis of CVD has been well established [60]. However, specific miRNAs associated with CVD in PCOS have not been as extensively described. MiRNA-339-5p was studied for its role in regulating endothelial progenitor cells (EPCs) in PCOS women [61]. EPCs are important in maintaining endothelial function, integrity, and neovascularization, as mature endothelial cells have a limited regenerative capacity [62]. EPC dysfunction is associated with endothelial dysfunction and is a common finding in PCOS patients that increases their risk for developing CVDs [61,62]. In the study, the upregulation of miRNA-339-5p was found to inhibit the migration, proliferation, and tubular formation of EPCs by inhibiting silent information regulator 1/peroxisome proliferator-activated receptor gamma coactivator 1-alpha (SIRT1/PGC-1α) and PI3K/AKT signaling pathways, making PCOS women more prone to developing CVD [61]. Findings were more evident in obese PCOS women included in the study [61]. Moreover, the study suggested the use of miRNA-339-5p as a promising potential therapeutic target to improve vascular endothelial health and prognosis in PCOS [61].

Further research needs to be done to fully understand the pathogenesis of CVD in PCOS in relation to miRNAs. However, it is thought that the reduction in vascular endothelial health is the primary driver of CVD in PCOS. MiRNAs can reduce the regenerative capacity of endothelial progenitor cells as well as worsen the metabolic profile of PCOS patients. This contributes to vascular damage and leads to a greater risk of developing CVD.

## 5. The Circadian Rhythm and PCOS

The circadian rhythm (CR) is crucial for regulating reproduction through the various functions of the hypothalamic-pituitary-gonadal (HPG) axis and the ovaries. A disordered CR affects reproductive outcomes by inducing insulin resistance, oxidative stress, and systemic inflammation [63]. The circadian clock network is composed of several transcription factors controlling a core feedback loop with transcription activator circadian locomotor output cycle kaput (*CLOCK*), brain and muscle Arnt-like protein 1 (*BMAL1*), clock genes Period (*Per1*, *Per2*, *Per3*), and Cryptochrome (*Cry1*, *Cry2*) [64].

These genes have a significant role in regulating metabolism and fertility. For instance, *BMAL1* plays a crucial role in lipid and glucose metabolism since it promotes adipogenesis [65]. In the hypothalamus, the suprachiasmatic nucleus (SCN) produces timing signals to activate gonadotropin-releasing hormone neurons, stimulating pituitary cells to release luteinizing hormone (LH) [66]. A study conducted on hamsters has found that long-term light exposure also desynchronizes the clock in the central and peripheral organs of the hamsters, which reduces pregnancy success rates [67].

**Figure 2:**
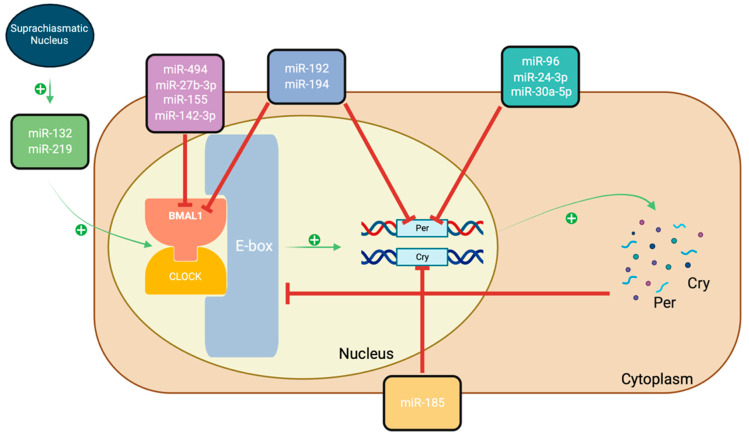
A schematic to illustrate the key genes and proteins involved in the circadian clock interacting with different miRNAs. *BMAL1* and *CLOCK* complex bind to E-box promoter elements, activating the transcription of *Per* and *Cry* genes. With a higher accumulation of genes in the cytoplasm, *CLOCK*:*BMAL1* activity is inhibited and prevents gene expression from acting as a negative feedback loop inhibitor [64]. Created with BioRender.com.

Many recent studies have demonstrated that miRNAs play a very crucial role in maintaining the CR (Figure 2). MiR-132 and miR-219 both displayed oscillations in the suprachiasmatic nucleus and are involved in activating the *CLOCK*:*BMAL1* complex. Recent literature suggests that miR-132 expression was significantly decreased in the follicular fluid of PCOS patients and is hypothesized to contribute to the pathophysiology of the syndrome by affecting *HGMA2* gene expression [68]. This gene was found to promote granulosa cell proliferation and was demonstrated to be activated in patients with PCOS [69]. Furthermore, miR-494, miR-27b-3p, miR-155, and miR-142-3p regulate *BMAL1* expression within the complex post-transcription. Recent case-control studies demonstrated that miR-27b, miR-155, and miR-142-3p expression were higher in PCOS patients [70,71,72]. MiR-192 and miR-194 have also been observed to regulate *BMAL1* post-transcription as well as inhibit the expression of the *Per* gene [73]. Mir-194 has been identified as a potential diagnostic marker in PCOS patients, as a case-control study demonstrated that it had a higher expression in PCOS patients [74]. Further studies have shown that miR-96, miR-24-3p, and miR-30a-5p directly target the *Per2* gene [75]. These microRNAs were found to have a higher expression in PCOS subjects within the serum fluid, follicular fluid, and rat ovaries, respectively [76,77,78]. *Cry1* translation is directly regulated by miR-185 [79]. Hence, the miRNAs involved in the CR should be further explored as possible targets for the treatment of PCOS.

Although it was demonstrated that miRNA-mediated post-transcriptional regulation regulates circadian oscillations, the CR itself can also regulate miRNA expression. Chen et al. demonstrated that the increased expression of *BMAL1* stimulates miR-103 expression in rats, leading to CR regulation [80]. Hence, although miRNAs and the CR interact with one another, their influence on the regulation of the biological clock is complex. This poses a challenge for researchers to fully grasp the mechanism of action and therapeutic potential of miRNAs within PCOS.

## 6. Potential Diagnostic and Prognostic Uses of miRNAs in PCOS

Multiple miRNAs have been found to be differentially expressed when comparing women with PCOS to healthy controls. This allows miRNAs to serve as potential biomarkers for PCOS diagnosis and prognosis, as they could help distinguish the different PCOS phenotypes as defined by the Rotterdam criteria (Table 4).

### 6.1. Biomarkers in Serum/Plasma

MiRNAs are present in abundance in serum and could be used as non-invasive biomarkers for PCOS as they are easy to detect, stable, and resistant to nuclease activity [81,82]. MiR-222, miR-146a, and miR-30c were found to be aberrantly expressed in the serum of PCOS women compared to controls [83]. MiR-222 was suggested as a useful marker of insulin sensitivity and T2DM as it positively correlated with serum insulin. In addition, there was a negative correlation between serum testosterone and miR-146a. These miRNAs were also found to target genes involved in metastasis, cell cycle, and apoptosis, which suggests a role in the pathogenesis of PCOS [83].

**Table 4:** MiRNAs as diagnostic and prognostic markers of PCOS.

miRNA(s)	Sample	Diagnostic/Prognostic Value	References
miR-222	Serum	Marker of insulin sensitivity and type 2 diabetes	[83]
miR-146a	Serum	Negatively correlates with serum testosterone	[83]
miR-29a-5p	Serum	Marker of PCOS	[84]
miR-320	Serum	Negatively correlates with insulin resistance	[84]
miR-339-5p	Serum	Marker of vascular endothelial health in PCOS	[61]
miR-103a-3p miR-21-5p miR-376a-3p	Serum	Positively correlate with androgen levels	[85]
miR-155	Serum	Monitor estroprogestinic treatment in hyperandrogenic PCOS women	[86]
miR-93	Plasma	Marker of PCOS	[31]
miR-146a-5p miR-126-3p	Plasma	Related to anovulation and polycystic ovaries	[87]
miR-20b-5p	Plasma	Potential marker of insulin resistance	[87]
miR-18a-3p	Plasma	Inversely correlates with luteinizing hormone	[87]
miR-106a-5p miR-20b-5p miR-18a-3p	Plasma	Correlates with plasma steroid hormones	[87]
miR-376a-3p miR-103-3p miR-139-5p miR-28-5p	Serum	Reflect androgenic profile	[88]
miR-92b miR-92a	Theca cells	Correlate with hyperinsulinemia/IR and hyperandrogenism profile	[45]
miR-423	Granulosa cells and follicular fluid	Downregulated, marker of PCOS	[89]
miR-142 miR-33b	Granulosa cells and follicular fluid	Upregulated, a marker of PCOS	[89]
miR-199b-5p	Follicular fluid	Correlates with anti-mullerian hormone	[39]
miR-382-5p	Follicular fluid	Correlates with free androgen index and age	[39]
miR-93-3p	Follicular fluid	Correlates with C-reactive protein	[39]
miR-490-5p miR-212-3p miR-4643	Follicular fluid and cumulus cells	Identification of PCOS subtype	[34]
miR-647	Follicular fluid and cumulus cells	Identification of PCOS subtype	[34]

Moreover, a meta-analysis found miR-29a-5p and miR-320 in the serum of PCOS women and suggested their use as biomarkers [84]. MiR-29a-5p had the most abnormal expression versus other miRNAs in the studies included in the meta-analysis and thus was suggested as a superior diagnostic biomarker. In addition, miR-320 was identified as a potential biomarker for insulin resistance [84]. Both miRNAs were found to be downregulated in PCOS patients compared to controls across different studies [84]. In a different study, several other miRNAs were shown to have a role in the pathways involved in steroid biosynthesis, folliculogenesis, endothelial regulation, and insulin signaling. The increased expression of miR-21-5p and the downregulation of miR-376a-3p and miR-103a-3p were found to be positively correlated with androgen levels [85].

Another study has demonstrated the use of serum miR-155 as a potential biomarker for monitoring estroprogestinic treatment in hyperandrogenic PCOS women [86]. Moreover, serum miR-339-5p was suggested as a marker of vascular endothelial health in PCOS [61], and plasma miR-93 could act as a useful biomarker for PCOS [31]. In addition, plasma exosomal miRNAs were also demonstrated as potential biomarkers [87]; the expression of miR-146a-5p and miR-126-3p in PCOS women was related to anovulation and polycystic ovaries while the expression of miR-106a-5p, miR-20b-5p, and miR-18a-3p was related to plasma steroid hormones. In addition, miR-20b-5p was suggested as a marker for insulin resistance, and an inverse relationship was found between miR-18a-3p and luteinizing hormone levels [87]. The altered levels of these miRNAs are thought to target functions such as axon guidance, circadian rhythm, and mitogen-activated protein kinase (MAPK) signaling pathways [87].

Likewise, certain miRNAs have been found to be altered during the course of follow-up of PCOS patients compared to healthy controls. While the control group had no changes in their miRNA during the follow-up, PCOS patients had twenty-six miRNAs that were altered in serum [88]. Four of these miRNAs were significantly decreased compared to healthy controls, including miR-376a-3p, miR-103-3p, miR-139-5p, and miR-28-5p. These miRNAs are predicted to be involved in several signaling pathways, including interleukin signaling, insulin signaling, and gonadotropin-releasing hormone receptor pathways. It was reported that PCOS patients had decreased levels of androgen during their follow-up, and miR-139-5p correlated with total testosterone levels. Androgen metabolism and miRNAs associated with PCOS decrease during follow-up and reflect a less hyperandrogenic profile in PCOS patients. Thus, it may be helpful to use these miRNAs as prognostic markers to reflect the androgenic profile of PCOS patients [88].

It is important to note that while levels of miRNA in the circulation may prove useful as biomarkers of disease, cautious interpretation must be exercised as their concentrations at the level of the tissue are, in many cases, not known.

### 6.2. Biomarkers in Theca Cells

MiR-92b and miR-92a are differentially expressed in theca cells. They are downregulated in patients with PCOS and are believed to augment signal transduction in the insulin and androgen pathways [45]. This significant downregulation in PCOS patients compared to controls suggests their utility as diagnostic biomarkers [45].

### 6.3. Biomarkers in Granulosa Cells

MiRNA expression in granulosa cells differs between PCOS patients and healthy controls. In PCOS patients, miR-423 is downregulated, while miR-142 and miR-33b are upregulated [89]. These miRNAs were also found to be dysregulated in follicular fluid. The aberrant expression of these miRNAs was shown to repress transforming growth factor beta signaling, repress apoptosis, and promote cell proliferation in cultures of granulosa cells and is believed to contribute to the pathophysiology of PCOS [89].

### 6.4. Biomarkers in Follicular Fluid

A recent study identified seven miRNAs in the follicular fluid that significantly differed in PCOS patients: miR-382-5p, miR-361-3p, miR-199b-5p, miR-381-3p, miR-93-3p, miR-127-3p, and miR-425-3p. MiR-199b-5p correlated with anti-mullerian hormone, miR-382-5p correlated with free androgen index and age, and miR-93-3p correlated with C-reactive protein [39]. These markers were suggested to be diagnostically and prognostically useful. In addition, women with PCOS had increased expression of miR-490-5p, miR-212-3p, and miR-4643 in follicular fluid and cumulus cells, with a decreased expression of miR-647 [34]. The altered expression profile of miRNA in follicular fluid and cumulus cells further suggests that miRNAs play a critical role in folliculogenesis as they regulate important pathways such as dehydroepiandrosterone (DHEA) metabolism and steroid biosynthesis. Therefore, these miRNAs could increase the risk for PCOS and serve as possible diagnostic markers for the identification of PCOS subtypes [34].

Overall, it is clear that miRNAs are potentially useful as minimally or non-invasive diagnostic and prognostic markers of PCOS. MiRNAs can be utilized to establish a diagnosis and help distinguish subtypes of PCOS. They could also aid in monitoring treatment and identifying the disease status in patients. Further research needs to be conducted to translate these findings into clinical practice.

## 7. Other RNAs in PCOS

### 7.1. The Role of Long Non-Coding (lncRNA) RNAs in PCOS

The two main categories of non-coding RNAs are miRNAs and lncRNAs. LncRNAs are non-coding RNAs that contain over 200 nucleotides and can either be circular or linear. They are crucial for various cellular processes, including gene regulation, transcription, chromatin modification, and epigenetic regulation [90]. By interacting as the competitive endogenous RNA (ceRNA), various lncRNAs have been shown to serve a crucial role in modulating granulosa cell proliferation and apoptosis. LncRNAs may regulate development, differentiation, proliferation, and apoptosis [91]. Furthermore, it has been shown that lncRNAs are expressed and have functional purposes in the follicular phase of the menstrual cycle [91]. This suggests a direct role of lncRNAs in the pathogenesis of PCOS. Moreover, lncRNAs can interact with miRNA, and certain lncRNAs can influence miRNA synthesis and activity. Associations between miRNAs and lncRNAs have a significant impact in modulating a wide range of biological processes, including development, nutrition absorption, and biotic and abiotic stresses [92].

#### 7.1.1. Effects of lncRNAs in PCOS

Long non-coding RNAs can act as miRNA decoys by sequestering miRNAs, thus acting as competing endogenous RNAs and leading to the re-expression of miRNA target genes [93] (Table 5). An important lncRNA associated with PCOS is lncRNA Cyclin L1 (CCNL1)-3:1, which was shown to be highly expressed in the granulosa cells of PCOS patients when compared to control groups [94]. This is thought to be attributed to the hyperandrogenic state in PCOS. In a study, lncRNA CCNL overexpression was observed when a human granulosa-like tumor cell line (KGN cells) was cultivated in various concentrations of dihydrotestosterone (DHT). Reactive oxygen species production was also greater, and ATP concentration was decreased in these cells, which is associated with follicular atresia in PCOS. This is believed to be due to the hindrance of mitochondrial function associated with lncRNA CCNL overexpression [94]. Furthermore, lncRNA CCNL contributes to the development of insulin resistance, which leads to the premature luteinization of granulosa cells [93]. When lncRNA CCNL was overexpressed, the number of glucose transporters, particularly insulin receptor substrate 1 (IRS1) and glucose transporter type 4 (GLUT 4), was reduced. In addition, overexpression of lncRNA CCNL is related to the elevated expression of forkhead box 01 (FOX01) at the protein level in KGN cells and human luteinized granulosa cells (hLGCs). FOX01 is a protein abundantly expressed in the ovary, adipose tissue, and endometrium. It causes cell cycle arrest, apoptosis, insulin resistance, and oxidative stress. It was shown that FOX01 knockdown reduced the impact of lncRNA CCNL overexpression on cell apoptosis and glucose transport, suggesting its importance in PCOS progression and identifying it as a target for the management of PCOS [94].

Moreover, the suppression or amplification of long noncoding granulosa-cells-differentiation-associated RNA (lncRNA GDAR) is thought to affect apoptosis in granulosa cells [94]. When control groups and high glucose groups were compared, it became clear that lncRNA GDAR modulates GC proliferation and apoptosis, which contributes to the growth of follicles. The outcome indicated that lncRNA GDAR was amplified in the low glucose group. By modulating the expression of mRNAs and proteins that are associated with apoptosis, such as caspase-3, caspase-7, B cell leukemia/lymphoma protein 2 (Bcl-2), B cell leukemia/lymphoma 9 (Bcl-9), bcl-2 associated x protein (Bax), lncRNA GDAR is known to prevent granulosa cells from apoptosis providing a protective effect. Therefore, lncRNA GDAR regulates follicle growth and positively affects the development of granulosa cells, which is found to be reduced in PCOS. However, this conclusion requires further mechanistic research [91].

Furthermore, a study indicated the overexpression of lncRNA BANCR and its involvement in insulin resistance. The study demonstrated that the expression of Bax protein levels in KGN cells was remarkably increased by the overexpression of lncRNA BANCR. This indicates that the apoptosis of KGN cells could be caused by the upregulation of Bax through lncRNA BANCR overexpression. Moreover, the expression of lncRNA BANCR in GCs and KGN cells was particularly elevated by treating insulin in a dose-dependent method. Therefore, the data in this study implied that lncRNA BANCR may be associated with pathological insulin signaling, leading to PCOS progression. Additionally, it could be a therapeutic target aimed at the prevention of insulin resistance in patients with PCOS [95].

Overall, lncRNA CCNL is elevated in luteinized granulosa cells and has a role in PCOS complications such as insulin resistance and follicular atresia by decreasing glucose transport capacity and promoting apoptosis. Follicular atresia is caused by oxidative stress due to elevated reactive oxygen species production, contributing to granulosa cell apoptosis. Additionally, insulin resistance and hyperinsulinemia lead to the premature luteinization of granulosa cells. lncRNA GDAR plays a role in modulating the expression of mRNAs and proteins associated with apoptosis and is thought to be reduced in PCOS. Meanwhile, lncRNA BANCR is upregulated in PCOS and is thought to affect cell growth.

#### 7.1.2. The Interaction between miRNAs and lncRNAs in PCOS

Interactions between lncRNAs and miRNAs have been demonstrated in the literature to have a crucial function in the pathogenesis of PCOS (Table 5). A recent study reports a role for LncRNA Zinc finger antisense 1 (lncRNA ZFAS1)/miR-129/high mobility group box 1 (*HMGB1*) axis in the endocrine disturbance in PCOS. It suggests that lncRNA ZFAS1 is upregulated and miRNA-129 is downregulated in PCOS, which leads to the overexpression of the *HMGB1* gene. This impacts the development of granulosa cells, resulting in hormonal disturbance and insulin resistance [96,97]. The higher levels of lncRNA ZFAS1 and *HMGB1* are also associated with an increased risk of ovarian malignancy. The study also proposes that the modulation of levels of these molecules could be protective in PCOS; however, not enough data are available to establish this conclusion. Additionally, the study suggests that silenced lncRNAs can be used for the prognosis and management of PCOS [96].

Moreover, lncRNA nuclear-enriched abundant transcript 1 (lncRNA NEAT1) and lncRNA metastasis-associated lung adenocarcinoma transcript 1 (lncRNA MALAT1) are long non-coding RNAs that are believed to regulate important miRNAs for normal endocrine function. In PCOS, lncRNA NEAT1 is overexpressed in serum and granulosa cells, which is associated with higher levels of insulin receptor substrate-2 (IRS-2), androgen receptor (AR), and follistatin (FST) expression via miR-30d-5p sponging [98]. This affects PCOS progression and further exacerbates PCOS symptoms [98]. Additionally, lower levels of lncRNA MALAT1 were observed in PCOS, which also contributed to the higher expression of the genes mentioned via the reduced regulation of miR-30a-5p activity [98].

Furthermore, the increased activity of lncRNA HOTAIR is related to higher expression of IGF1 in PCOS. This effect is thought to be exerted by competitively inhibiting miR-130a, which was observed in PCOS rat models [98]. The higher activity of IGF1 is associated with disturbances in ovarian function, elevated androgen production, and cancer. The study suggests that the downregulation of lncRNA HOTAIR and upregulation of miR-130a could be potentially therapeutically beneficial in PCOS. However, other organ systems might be affected by this intervention, which requires additional investigation [99]. Moreover, a study demonstrated that the upregulation of lncRNA HCP5 in PCOS patients had a similar effect on IGF1 through the competitive inhibition of miR-27a-3p; thus, it can also be utilized as a therapeutic target [100].

In summary, modulating interactions between lncRNAs and miRNAs can be therapeutically useful in PCOS. Upregulated lncRNA ZFAS1 and downregulated miR-129 levels are associated with insulin resistance and hormonal disturbance, which is mediated by higher expression of HMGB1 protein. In addition, both lncRNA NEAT1 and lncRNA MALAT1 are involved in the regulation of different miRNAs, which are responsible for normal endocrine functioning and are both abnormally expressed in PCOS. Meanwhile, lncRNA HOTAIR acts as a competitive inhibitor of miR-130a. Similarly, lncRNA HCP5 competitively inhibits miR-27a-3p, which leads to increased expression of IGF1. Using receiver operating characteristic curve analysis, the effective use of lncRNAs (specifically lncRNA NEAT1 and lncRNA MALAT1) as diagnostic markers has been shown, with most diagnostic utility shown using a combination of lncRNA NEAT1 and miR-30d-5p, or lncRNA MALAT1 and miR-30a-5p with PCOS target genes [98]. The findings indicate the use of lncRNAs as biomarkers for the diagnosis of PCOS and present possible therapeutic targets for the management of PCOS. However, further research is necessary to verify the implications of lncRNAs in the clinical management of PCOS.

**Table 5:** LncRNAs in PCOS.

lncRNA	Role in PCOS	References
Effects of lncRNAs in PCOS		
lncRNA CCNL1-3:1	Overexpression of lncRNA CCNL elevates FOX01 expression, resulting in cell death, decreased glucose transporters, and altered mitochondrial functions. Therefore, lncRNA CCNL leads to two main complications of PCOS, which are follicular atresia and insulin resistance.	[94]
lncRNA GDAR	lncRNA GDAR is reduced in PCOS, leading to a greater expression of mRNAs and proteins associated with the apoptosis of granulosa cells.	[91]
lncRNA BANCR	LncBANCR is upregulated in PCOS, enhancing cell death and preventing cell growth, indicating a role in the progression of PCOS.	[95]
Interactions between lncRNAs and miRNAs in PCOS		
lncRNA ZFAS1	lncRNA ZFAS1 is increased, and miR-129 is reduced in PCOS patients. The interactions of lncRNA ZFAS1 and miR-129 induce PCOS by increasing apoptosis of granulosa cells via increased *HMGB1* gene expression.	[96]
lncRNA NEAT1 and lncRNA MALAT1	In PCOS, lncRNA NEAT1 has elevated levels in serum and granulosa cells; however, lncRNA MALAT1 is repressed. Their involvement in PCOS development relies on adjusting miR-30d-5p and miR-30a-5p, which affects IRS-2, AR, and FST expression.	[98]
lncRNA HOTAIR	Upregulated lncRNA HOTAIR competitively binds to miR-130a, leading to higher expression of IGF1 seen in rat models of PCOS.	[99]
LncRNA HCP5	LncRNA HCP5 is upregulated in PCOS, which competitively inhibits miR-27a-3p, leading to increased activity of IGF1.	[100]

### 7.2. The Role of Circular RNA (circRNA) in PCOS

Circular RNAs (circRNAs) are covalently closed continuous loops that have a tissue-specific role in the pathogenesis of a number of illnesses, including cancer, cardiovascular disease, and neurological conditions [101]. CircRNAs have a significant impact on PCOS (Table 6); their primary function is to modulate gene expression by controlling transcription and translation by sponging RNA binding proteins and/or miRNA [101].

A study that included 45 PCOS patients and 45 healthy controls revealed that circRANBP9, a non-coding RNA on chromosome 6, is elevated in PCOS patients [101]. Moreover, circRANBP9 acts as a sponge to miR-136-5p and is found elevated in plasma and granulosa cells. In accordance with the research, circRANBP9 loss lowers granulosa cell development and increases apoptosis by regulating miR-136-5p, which suggests a potential therapeutic use for PCOS. However, further investigations are required to fully delineate the role of circRANBP9 in PCOS progression [101].

Furthermore, another study in 22 patients with PCOS and 22 healthy controls showed circPMSC3 to be reduced in PCOS [102]. Additional research into the in vivo effects of overexpressed circPMSC3 was performed using a mouse model and demonstrated a reduction in insulin release induced by the glucose and normalization of ovarian tissue structure, suggesting that circPMSC3 may be critically important in PCOS. In addition, the overexpression of circPMSC3 increases the levels of phosphatase and tensin homolog (PTEN). Using a mouse model of PCOS, circPSMC3 was found to relieve the symptoms through sponging miR296-3p, which boosts PTEN expression [102].

Additionally, research on circRHBG, which is overexpressed in PCOS, was conducted by comparing the viability of KGN cells and SV40-transfected, immortalized human granulosa cell line (SVOG cells) with various levels of circRHBG expression [103]. This showed that suppressing circRHBG levels impeded KGN and SVOG cell growth significantly, demonstrating that circRHBG is a crucial factor in PCOS development and could potentially be used as a diagnostic and treatment target for PCOS. circRHBG prevents ferroptosis, which results in cell death by accumulating lipid peroxidation products and ROS. Therefore, circRHBG contributes to the progression of PCOS by reducing cell proliferation and inhibiting ferroptosis [103].

Circ-FURIN, which is found on chromosome 15 and amplified in PCOS, was shown to be more prevalent in KGN cells among PCOS women [104]. Reduction of circ-FURIN elevated caspase-3 and Bax levels and lowered p-PI3K levels. CircFURIN additionally elevated BCL2 expression by sponging miR-195-5p. Through the miR-195-5p/BCL2 axis, the loss of circFURIN impaired cell proliferation and enhanced apoptosis in KGN cells, leading to the progression of PCOS [104].

Studies have revealed that circPUM1, another circRNA that acts as a sponge to mi-R-760, is additionally involved in the development of PCOS. MiR-760 suppression promotes cell growth and prevents cell death. As a result, the expression of circPUM1 advances disease through apoptosis [105]. Moreover, in a study investigating the pathology of PCOS using the human granulosa-like tumor KGN cell line [105], circASPH was found to be elevated in PCOS, reducing the growth of KGN cells and promoting their death. Furthermore, circASPH was shown to play an oncogenic role in the etiology of PCOS [106].

In PCOS GC and KGN cells, the upregulation of circ_0043532 was demonstrated. Furthermore, silencing circ_0043532 caused the suppression of cell growth and stimulated cell death and cell cycle arrest by regulating the miR-182/SGK3 axis in GCs and KGN cells of patients with PCOS. The findings suggest that the suppression of circ_0043532 reduces the speed of PCOS progression and further suggests a therapeutic target for PCOS patients [107]. Moreover, a recent study has demonstrated the involvement of circMTO1 in the pathogenesis of PCOS. CircMTO1 was found to mediate the progression of human granulosa-like tumor cells through the miR-320b/MCL1 axis, promoted by increased transcription of SNAI2. Thus, Duan et al. concluded that a SNAI2/circMTO1/miR-320b/MCL1 axis is a promoter of human granulosa-like tumor cells in PCOS [108].

Additionally, one study reported finding a higher expression of circ_0030018 in PCOS patients. The pathway for this circRNA involves miR-136, which further expresses MEIN1, leading to AKT activation. It has been determined that circ_0030018 promotes the growth of several tumors, and targeting it would reduce tumor growth. As such, suppressing this circRNA in PCOS patients is believed to help alleviate symptoms of PCOS. This pathway’s role is understood in terms of tumor growth but less so as regards the etiology of PCOS. However, Xu et al. concluded that this pathway is significant to PCOS pathology, and targeting it therapeutically would alleviate symptoms of PCOS [109].

Overall, circRNAs that have a role in the development of PCOS, including circRANBP9, circFURIN, circRHBG, circASPH, circPUM1, circ_0043532, circMTO1, and circ_0030018 were shown to be elevated in women with PCOS. These circRNAs have a role in the development of PCOS and could act as possible diagnostic markers and therapeutic targets for the disease. Further research needs to be undertaken to understand how these RNAs could be effectively utilized clinically.

## 8. Future Directions

The existing literature exploring the relationship between PCOS and miRNA, lnRNAs, and circRNAs is limited. Further studies may investigate the following:

- Establish a definitive set of miRNAs that are common in the majority of PCOS patients, which can be used as prognostic markers and for disease monitoring purposes.
- Further research to identify the mode of function of miRNAs to understand how they manipulate and are manipulated by insulin, thereby providing potential therapeutic targets not only for PCOS patients but all those suffering from insulin disorders (such as T2DM, pre-diabetes, etc.).
- Previous studies often do not clearly define the PCOS population in terms of the Rotterdam criteria, and therefore, comparisons are often of mixed phenotypes. Consequently, the results can be difficult to interpret.
- Studies to identify potential therapeutic uses of miRNA in PCOS patients.
- Further research is needed to establish reliable, non-invasive methods to diagnose PCOS.
- Studies to identify the potential for MiR-143-3p and MiR-155-5p to act as treatment targets in PCOS patients.
- Further studies are needed to identify the relationship between CVD and PCOS in relation to miRNA, as there is a lack of literature surrounding this area.
- Studies to investigate lncRNAs as circulating biomarkers for PCOS and their potential to monitor treatment effectiveness.
- As various circRNAs have been associated with the progression of PCOS, studies may identify potential diagnostic and therapeutic uses of circRNAs.

## 9. Conclusions

PCOS is a complicated heterogeneous condition with an unclear etiology, and the appropriate diagnosis and management of PCOS remains a challenge for healthcare professionals. Growing evidence suggests that miRNAs are involved in key regulatory pathways associated with the pathogenesis of PCOS and the interplay with obesity. Dysregulated miRNAs can affect granulosa and theca cells, vascular endothelial cells, and adipocytes, leading to complications such as infertility, insulin resistance, and cardiovascular disease in PCOS. These regulatory networks can be extremely complex, and some examples of the miRNAs affecting the above-mentioned regulation processes include miR-27a-3p, miR-323-3p, miR-155-5p, and miR-339-3p. This also suggests a use for miRNAs as diagnostic and prognostic markers as well as therapeutic targets for the disease. Furthermore, miRNAs play a vital role in maintaining the circadian rhythm, which is crucial for regulating reproduction. Hence, these miRNAs can be further explored as possible molecular treatment targets. MiRNAs interact with other RNAs, notably circRNAs and lncRNAs, promoting the development of PCOS, and modulation of these interactions could be another therapeutic avenue. However, there are several inconsistencies in the literature regarding the miRNAs believed to play a significant role in PCOS. This may be due to multiple factors, including the different methodologies implemented in the various reports. Another factor may be the different ethnicities of the women studied, causing inconsistent results between populations. This suggests that selected miRNAs should be investigated further to ascertain their role in the regulatory processes of PCOS. In addition, the relationship between CVD, PCOS, and miRNAs needs to be investigated further, as there is a paucity of updated literature addressing this topic. Overall, it is evident that miRNAs play a significant role in the progression of PCOS and have great potential to be utilized in clinical settings. However, research on miRNA and PCOS is still in its relative infancy, although early indications appear promising.

## Figures and Tables

**Table 1:** Phenotypes of PCOS based on Rotterdam Criteria [12].

Phenotype	Androgen Excess	Ovulatory Dysfunction	Polycystic Ovarian Morphology on Ultrasound
A [Classic PCOS]	X	X	X
B [Essential NIH criteria]	X	X	
C [Ovulatory PCOS]	X		X
D [Non-hyperandrogenic PCOS]		X	X

**Table 2:** MiRNAs involved in the pathogenesis of infertility in women with PCOS.

miRNA(s)	Sample	Finding in PCOS	References
miR-18b	Follicular fluid	Upregulated, promotes the release of progesterone while inhibiting the release of estradiol and testosterone, thus negatively impacting fertility.	[41]
miR-146a	Follicular fluid	Upregulated, reduces progesterone, estradiol, and testosterone release, causing irregularities in the menstrual cycle.	[41]
miR-135a	Follicular cells	Upregulated, reduces progesterone and testosterone release	[41]
miR-93 miR-21	Granulosa cells	Upregulated in hyperandrogenic patients and has a role in follicular dysfunction.	[42]
miR-27a-3p	Granulosa cells	Impacts relative estrogen deficiency, which is believed to induce granulosa cell apoptosis.	[43]
miR-320a	Cumulus granulosa cells	Downregulated, has an effect on the IGF-1 regulatory mechanism and estrogen synthesis by targeting the *RUNX2* gene.	[44]
miR-92a miR-92b	Theca cells	Downregulated, play a role in hyperandrogenism in PCOS by regulating *CYP17*, *GATA6*, and IRS-2 gene expression.	[45]
miR-323-3p	Cumulus granulosa cells	Downregulated, promotes apoptosis, and upregulates steroidogenesis in cumulus cells.	[43]

**Table 6:** CircRNAs in PCOS.

CircRNA	Role in PCOS	References
circRANBP9	circRANBP9 is upregulated in PCOS patients. The loss of circRANBP9 reduces granulosa cell growth and promotes apoptosis via the regulation of miR-136-5p.	[101]
circPMSC3	circPMSC3 is downregulated in PCOS. Overexpression of circPMSC3 modulates miR296-3p that targets PTEN, which could have therapeutic use in PCOS.	[102]
circRHBG	circRHBG is upregulated in PCOS; it prevents cell proliferation and inhibits ferroptosis.	[103]
circFURIN	circFURIN is upregulated in PCOS; reduction of circFURIN impairs cell proliferation and promotes apoptosis.	[104]
circPUM1	circPUM1 functions as a sponge for miR-760, causing PCOS progression through apoptosis.	[105]
circASPH	circASPH is upregulated in PCOS; it restricts cell proliferation while promoting cell death and has an oncogenic role in the pathogenesis of PCOS.	[106]
circ_0043532	circ_0043532 is upregulated; silencing circ_0043532 leads to the suppression of cell proliferation and stimulated cell apoptosis, indicating it as a therapeutic target in PCOS.	[107]
circMTO1	Upregulated by SNAI2 and expressing miR-320b/MCL1 downstream, it aids the progression of human granulosa-like tumor cells.	[108]
circ_0030018	Upregulated in PCOS patients and expresses the miR-136/MEIN1 axis, causing elevated AKT in the cytoplasm.	[109]

## Abbreviations

Ago2	Argonaute RISC catalytic component 2
Bax	Bcl-2 associated x protein
Bcl-2	B cell leukemia/lymphoma protein 2
Bcl-9	B cell leukemia/lymphoma 9
BMAL1	brain and muscle Arnt-like protein 1
CCNL1	LncRNA Cyclin L1
ceRNA	Competitive endogenous RNA
circRNA	Circular RNA
circRNAs	Circular RNAs
CLOCK	Circadian locomotor output cycle kaput
Cry1, Cry2	Cryptochrome
CYP17	17-hydroxylase/C17–20 lyase cytochrome P50
FOX01	Forkhead box 01
Fox12b	Forkhead transcription factor 12b
GATA6	GATA-binding factor 6
Gdf	Growth differentiation factor
hLGCs	Human luteinized granulosa cells
HMGB1	High mobility group box 1
IGF-1	Insulin-like growth factor 1
KGN cells	Human granulosa-like tumor cell line
lncGDAR	Long noncoding granulosa-cells-differentiation-associated RNA
MALAT1	Metastasis-associated lung adenocarcinoma transcript 1
MAPK	Mitogen-activated protein kinase
NEAT1	Nuclear-enriched abundant transcript 1
NFkB	Nuclear factor kappa B
Per1, Per2, Per3	Period
PI3K/AKT(PKB)	Phosphoinositide-3-kinase/protein kinase B
pri-miRNA	Primary miRNA
pre-miRNA	Precursor miRNA
PTEN	Phosphatase and tensin homolog
SIRT1/PGC-1α	Silent information regulator 1/peroxisome proliferator-activated receptor gamma coactivator 1-alpha
TRBP	Dicer-transactivation response RNA binding protein
ZFAS1	Zinc finger antisense 1
